# The severity of perceived stress associated with community violence exposure and its role in future posttraumatic stress: findings from a longitudinal study of U.S. adolescents

**DOI:** 10.1186/s13034-024-00813-0

**Published:** 2024-09-19

**Authors:** Johan Isaksson, Sara Nyman, Mary Schwab-Stone, Andrew Stickley, Vladislav Ruchkin

**Affiliations:** 1 Department of Medical Sciences, Child and Adolescent Psychiatry Unit, Uppsala University, Uppsala, Sweden; 2 Center of Neurodevelopmental Disorders (KIND), Centre for Psychiatry Research, Department of Women’s and Children’s Health, Karolinska Institutet & Stockholm Health Care Services, Region Stockholm, Sweden; 3 Child Study Center, Yale University School of Medicine, New Haven, Connecticut USA; 4 Stockholm Centre for Health and Social Change (SCOHOST), Sodertorn University, Huddinge, Sweden; 5 Department of Preventive Intervention for Psychiatric Disorders, National Institute of Mental Health, National Center of Neurology and Psychiatry, Tokyo, Japan; 6 Sala Forensic Psychiatric Clinic, Sala, Sweden

**Keywords:** Trauma, Violence, PTSD, Stress, Sex

## Abstract

**Background:**

Community violence exposure (CVE) in adolescence is associated with poorer mental health although the situational factors that may impact on this relationship remain relatively unexplored. The present study aimed to investigate if the degree of perceived stress during CVE has an effect on future posttraumatic stress disorder (PTSD) symptoms in youth, and if this association differs between females and males.

**Methods:**

Data were analyzed from 760 U.S. adolescents (mean age = 14.00 years), who participated in two surveys, one year apart. Information about CVE (witnessing violence and violence victimization) and the stress severity associated with it was collected in the first survey, whereas data on PTSD symptoms were obtained in the second survey. Generalized Linear Models were used to examine the associations that were also adjusted for ethnicity, age and socioeconomic status.

**Results:**

The results showed a longitudinal association between CVE-related stress and future PTSD symptoms, with students who had higher levels of perceived stress during CVE (witnessing or victimization) reporting higher PTSD symptom levels subsequently. There was no interaction between sex and CVE-related stress and PTSD symptoms, although females reported more PTSD symptoms.

**Conclusions:**

The severity of the perceived stress associated with CVE should be regarded as a potentially important prognostic factor for identifying those who might develop PTSD symptoms after CVE and this may facilitate early intervention and treatment for those exposed to community violence.

## Background

Community violence exposure (CVE) is one of the most common adverse childhood experiences [1] and is defined as exposure to a violence-related act within one’s home, school, or neighborhood between individuals who are unrelated, and who may or may not know each other [2]. In a meta-analytic study, CVE in children and adolescents was linked to a wide range of mental health problems, including both internalizing (e.g., depression and anxiety) and externalizing (e.g., deviant and aggressive behavior) problems, both cross-sectionally and longitudinally [3]. That same study suggested that of all potential negative outcomes, CVE had the strongest association with posttraumatic stress disorder (PTSD) [3], a disorder characterized by exposure to a traumatic event with intrusive re-experiencing and avoidance of stimuli associated with that event, as well as negative changes in cognition, mood, reactivity, arousal and behavior [4]. The finding of an association between CVE and PTSD was further corroborated in a review on CVE and mental health outcomes in urban adolescents, where PTSD symptoms had one of the strongest relationships with CVE (which remained even when controlling for the other mental health variables and confounders) [5], and also in another study on African American adolescents, where CVE was most strongly and consistently associated with PTSD [6].

When trying to assess the associations between CVE and mental health outcomes such as PTSD it is important to recognize that the negative outcomes associated with CVE may vary depending on the degree of exposure, or physical proximity of children to a violent event, and the subjective severity of the associated stress. With regard to physical proximity, two main types of CVE have been identified: witnessing (the eye-witnessing of an event without being an actual victim oneself) and victimization (being the object of an intentional act of violence initiated by another person to cause one harm) [3, 7–9]. The negative outcomes experienced by children tend to increase in relation to their physical proximity to a violent event, where direct victimization generally produces stronger negative consequences, including more psychiatric symptoms, than witnessing, which in turn, has a greater effect than indirect exposure [3, 10, 11].

Having said this, in recent decades there has been a shift in the trauma perspective away from the objective nature of the stressor to the subjective experience of victimization, thus focusing on the victim, who must have experienced, witnessed or been confronted by a threat to physical well-being and responded as a result with intense fear, helplessness, or horror [12]. In relation to this, it has been demonstrated that the majority of stressors within the PTSD criteria involve a subjective emotional response [12]. Global symptomatic distress after injury [13], and in particular, a perceived threat to life [14], have been associated with an increased risk for the development of PTSD symptoms. Subsequent research has further demonstrated that subjective stress severity (i.e., the level of perceived stress during exposure to a stressor) is generally a stronger predictor of negative health outcomes than measures of stressor exposure (e.g., the occurrence of one or more stressors [15]). Hence, a focus on individual perceptions of stress severity would seem to be crucial, as the exposed person is arguably the best source of information for both determining the degree to which an event was stressful (e.g [16]), and for describing the circumstances that influence the degree to which an event had a negative impact on their life [17].

At the same time, previous research on the relation between perceived stress severity, CVE and PTSD has been limited to general measures of perceived stress which have not been assessed in response to the traumatic event itself [18–22]. Research exploring the role of perceived stress severity experienced during CVE in children and adolescents has also been limited. There is however, some research which suggests that a focus on CVE-related stress and PTSD may be warranted. An earlier cross-sectional study on PTSD in South African children found a positive correlation for both CVE and perceived stress with PTSD symptoms [18]. In addition, cross-sectional studies undertaken in adult populations have similarly found positive correlations between perceived stress and PTSD, including in female victims of violence in the U.S [19], oral cancer patients in China [20], and civil citizens exposed to terrorist attacks in Israel [21].

It is also important to consider the potential role that gender might play in any associations. Previous research has indicated that both boys and girls have an increased risk for mental health problems as they transition into adolescence [23]. As stressful life events tend to predict later mental health problems [4], it has been suggested that an increase in the prevalence of psychopathology observed in adolescence may be partially explained by the increase in the number of stressful life experiences during this developmental period (e.g [23– 25]). Further, some research has even indicated that the discrepancy in the types of problems observed between boys and girls in adolescence may be related to differences in how they perceive stressors [25, 26], and in particular, those stressors that are perceived as most threatening. For both boys and girls, CVE tends to have a particularly strong negative impact on the trajectory of psychopathology over time [3], and yet, although male adolescents are more often exposed to violence, females who experience trauma report more distress and impairment compared to traumatized males and are more often diagnosed with PTSD [27–29].

To summarize, the role of perceived stress severity in relation to the association between CVE and PTSD is not fully understood and thus requires further research. Moreover, to the best of our knowledge, as yet, there has been no longitudinal research on perceived stress that is directly associated with CVE and its potential impact on future PTSD symptoms in an adolescent population. Thus, in order to address this deficit, the present study will use longitudinal data in order to investigate if CVE-related stress at wave 1 is associated with PTSD symptom levels one year later at wave 2. The study also aims to investigate if this association differs between male and female adolescents. It is hypothesized that a higher level of CVE-related stress will generate more subsequent PTSD symptoms, and that this association will differ between the sexes.

## Methods

### Participants

This longitudinal study included 760 children aged 13–16 years old (mean age = 14.00, SD = 0.66), enrolled in the public school system in New Haven, Connecticut, a city located in the northeast US that has 134,000 inhabitants. A large proportion of the city’s residents are ethnic minority groups with a low socioeconomic status (SES). At wave 1, where the data collection took place during the fall of 2003, 1278 students completed the first survey. At the time of the follow-up survey one year later, 490 of the students had left the public school system and were therefore excluded from this study, and two students were excluded for being outside the study age range. Further, 26 of the remaining students did not provide the relevant data in the follow-up survey and were thus also excluded. No differences were observed for the study variables between those 26 students who were excluded and the 760 who were included in the study: witnessing CV (*χ2* = 3.56, *p* = 0.313), being victimized by CV (*χ2* = 1.25, *p* = 0.742), age (t = 1.22; *p* = 0.233), SES (*χ2* = 3.47, *p* = 0.177), sex (*χ2* = 2.81, *p* = 0.094) or race (*χ2* = 5.88, *p* = 0.208). The demographic characteristics of the study participants are presented in Table 1.

**Table 1:** Demographic characteristics of the study participants by sex, assessed with Chi-square tests and an independent sample t-test

	All	Females	Males	Statistics
	760	390 (51.3%)	370 (48.7%)	
Ethnicity, *n* (%)				
African American	481 (63.3%)	246 (63.1%)	235 (63.5%)	
Hispanic	182 (23.9%)	96 (24.6%)	86 (23.2%)	
White	82 (10.8%)	45 (11.5%)	37 (10.0%)	
Other	15 (2.0%)	3 (0.8%)	12 (3.3%)	*χ2* = 8.56^ns^
Age, M (SD)	14.00 (0.66)	14.00 (0.67)	14.00 (0.65)	*t* = 0.57^ns^
Socioeconomic status, *n* (%)				
Full price	279 (36.7%)	139 (35.6%)	140 (38.2%)	
Reduced lunch cost	88 (11.6%)	37 (9.5%)	51 (13.8%)	
Free lunch	393 (51.7%)	214 (54.9%)	179 (48.4%)	*χ2* = 4.83^ns^

*M* Mean, *ns* non-significant, *SD* Standard deviation

### Procedure

The data in this study come from a large-scale longitudinal study that examines problem behavior in middle and high school students, and the risk and protective factors for such behavior. All children in grade eight attending a public school in New Haven, Connecticut were asked to participate. Parents received information about the study at the time of the school registration and were also informed about the survey in a letter two weeks before it took place, with the possibility to deny their child’s participation. Before the students took the survey, they were informed about the study and assured that it was confidential, and they then provided informed consent to participate. Specifically, the participating students signed a consent form on the first page of the survey, which was then removed, and the rest of the survey, including the questionnaires, were anonymized with a code. Although students could decline participation, less than 1% of the students decided not to participate. The students answered two surveys across two waves one year apart. The surveys were completed by the students in their classrooms during a normal school day. Each question was read out aloud by a trained administrator, with students following along using their personal copies of the survey questionnaire and then marking their answers. For this study, the data on CVE and the perceived stress related to it were obtained in the first survey, whereas the data on PTSD symptoms were obtained in the second survey. Information on the students’ sex, age, ethnicity, and SES (i.e., paying or not paying a reduced price for school lunch) was retrieved from the school registry. Ethical permission for the study was provided by the institutional review board at the Yale University School of Medicine.

### Measures

#### Community violence exposure and associated perceived stress

Fourteen questions were used to assess the witnessing of and being victimized by community violence (CV) i.e., 7 questions were used for each type of CVE that were derived from the Screening Survey of Exposure to Community Violence [30]. That measure has been used in numerous studies, particularly in research focused on urban populations where community violence is a significant concern [3], and has been shown to have good internal consistency and test-retest reliability [31]. It has also been validated by mapping the location of the actual homicide cases in New Haven, CT to the zip codes of the students reporting on CVE, showing a high degree of agreement [32]. The students provided information about whether or not they had witnessed and/or been a victim of seven different CV events in the past year (e.g., chased by gangs or individuals, being/seen someone else seriously wounded after an incident of violence, threats of physical harm, muggings or beatings, violence involving guns and knives). The students answered using a 5-point response format, ranging from ‘Never’ to ‘10 or more times’. The items for each measure were combined so that there were separate witnessing and victimization variables with total scores. While each of these variables represent an index rather than a scale (i.e., being exposed to one event doesn’t automatically imply a greater chance of being exposed to another one), a high degree of internal consistency (Cronbach’s α = 0.88 for each scale) suggests that multiple CV events were often experienced by the same individuals. This would make it difficult to disentangle the individual effect of each particular event and these witnessing and victimization scale scores were then recoded into dichotomous variables, indicating whether the respondent had or had not experienced each type of CVE. If the student had witnessed one or more violent events, they were coded as having witnessed CVE. Similarly, if the student had been a victim of one or more violent events, they were coded as having been a victim of CVE. This dichotomizing of the scale scores corresponds with the use of these measures in several previous studies, e.g [33, 34]. In addition, for each CVE event that the students had experienced, they answered a follow-up question inquiring about the level of perceived stress severity they had experienced in relation to that particular event (“how much did it bother you the last time it happened?”), using a five-point Likert scale: no stress, a little stress, some stress, a moderate amount of stress, and a lot of stress.

For the purposes of the present study, the information on the level of CVE-related stress was recoded into perceived severity of stress scores, calculated for witnessing and victimization separately. For witnessing, those who reported not having witnessed any CV event (i.e., answering never) were coded as “0”, those who had witnessed CV (i.e., 1 or more times) but experienced no stress in relation to the exposure were coded as “1”. Those who had witnessed CV and experienced a little or some stress were coded as “2”, and those who had witnessed CV and experienced a moderate amount or a lot of stress were coded as “3”. For victimization the recoding was done in the same way. It should be noted that as students could report experiencing more than one event of witnessing and/or victimization and the related stress ratings could differ depending on the specific event, in this study we used the highest levels of stress reported in relation to a CVE event to categorize the overall stress rating. The possible CVE-related stress scores for witnessing and for victimization could thus range between 0 (no CVE) and 3 (CVE and a moderate amount or a lot of stress), with higher scores indicating a higher level of perceived stress severity associated with each type of CVE.

#### SES

In the U.S. government supported programs that allow reduced lunch fees or free lunches to be provided are administered by schools in order to assist families with lower income levels. Information about the students’ eligibility for subsidized school lunches was obtained from the school register, with three possible price options: paying the full price (scored as 0), paying a reduced price (scored 1) or receiving a free lunch (scored 2). This information was then used as a proxy for SES, with higher scores indicating worse SES. At the time of the data collection, students whose family income was 185% or less of the federal poverty line were eligible for free or reduced lunches. The use of information about the students’ eligibility for subsidized school lunches as a proxy measure for SES has occurred in previous studies, e.g [33].

#### Posttraumatic stress

The 20-item Child Post-Traumatic Stress Reaction Index (CPTS-RI) was used to assess posttraumatic stress in this study [35]. This scale was originally developed to measure the syndromic components of the earlier version of the Diagnostic and Statistical Manual of Mental Disorders (DSM-III-R) criteria for PTSD, and although the included symptoms are very similar to the DSM-IV criteria with re-experiencing, avoidance/numbing and arousal symptoms, the scale does not include all the symptoms described in DSM-5. Specifically, apart from the criterion of exposure to a traumatic event, which the CPTS-RI does not evaluate, DSM-5 has more behavioral symptoms accompanying PTSD, and proposes four distinct diagnostic clusters instead of the previous three, including altered cognitions and moods. Despite this, the CPTS-RI is still one of the most widely used tools to assess symptoms of posttraumatic stress in children and adolescents after a traumatic exposure, and has been consistently found to have a good degree of reliability, a high level of internal consistency and moderate test–retest consistency [36, 37]. The frequency of PTSD symptoms in the last four weeks is assessed using a five-point Likert-type scale where the response options range from never (scored 0) to most of the time (4). The Index has a total score of 80 points, and correlates well with the diagnosis of PTSD in the DSM nomenclature [30]. A total score of 60 points or higher indicates very severe posttraumatic stress levels, 40–59 points - severe levels, 25–39 - moderate levels, and 12–24 mild levels. For this study, the CPTS-RI ratings were used as a continuous score. Cronbach’s α for this measure was 0.87.

### Statistical analyses

The data analyses were performed with SPSS version 28. The outcome variable (PTSD symptoms) had a right-skewed gamma distribution (skewness = 1.18, SE = 0.09; kurtosis = 1.11, SE = 0.18) and hence was not normally distributed. The Kruskal-Wallis one-way analysis of variance test was used for the CVE and degree of perceived stress group comparisons in relation to PTSD symptoms, with a Bonferroni correction being applied. The Mann-Whitney U test was used for analyzing sex differences in relation to PTSD symptoms, and Chi-square tests were employed to assess CVE ratings. Spearman’s rho was used to assess the correlations between the measures of CVE-related stress for the two types (witnessing and victimization) of CVE. For the main analyses, Generalized Linear Models (GLM) were used. The Akaike information criterion (AIC) indicated that a gamma distribution with a log link function had the best model fit with the lowest AIC value (5849.19). Therefore, GLM with Gamma as the distribution and Log as the link function were used in order to assess differences in PTSD symptoms at wave 2 in males and females, in relation to different levels of CVE-related stress (no CVE (0), CVE and no perceived stress (1), CVE and a little or some stress (2), and CVE and moderate or a lot of stress (3)). Two models were fitted, one for witnessing and one for victimization. The analyses were adjusted for sex, ethnicity (with separate dummy variables created for African-American (1/0) and Hispanic (1/0) ethnicity), age and SES, and an interaction of sex by CVE-related stress on PTSD symptoms was calculated in separate models. A p-value < 0.05 was considered statistically significant.

## Results

### Group comparisons

Self-assessed PTSD symptom scores in relation to the amount of perceived stress when witnessing or being a victim of CV, are presented in Table 2 for the total sample and by sex. PTSD symptoms at wave 2 differed between the CVE-related stress groups, both in relation to witnessing (H(3) = 60.55, *p* < 0.001) and victimization (H(3) = 40.88, *p* < 0.001). The results from the Kruskal-Wallis pairwise tests adjusted with a Bonferroni correction, showed that among the total sample for witnessing those who reported a moderate amount or a lot of stress at wave 1 had higher PTSD symptoms levels at wave 2 than those reporting no witnessing of CV (*p* < 0.001), no stress in relation to CVE (*p* < 0.001), or a little or some stress from CVE (*p* < 0.001). Further, those who reported a little or some stress (*p* = 0.032) had more PTSD symptoms than those reporting no witnessing of CV. For victimization, Kruskal-Wallis pairwise tests adjusted with a Bonferroni correction showed that for the total sample both those who experienced a little or some stress (*p* = 0.008) and those who experienced a moderate amount or a lot of stress (*p* < 0.001) had higher PTSD symptom levels compared to those with no exposure. Compared to males, females reported higher PTSD symptom levels (*U* = 53484; *p* < 0.001). More females than males said they had witnessed CV and experienced a moderate amount or a lot of stress (*χ2* = 26.04, *p* < 0.001), whereas more males than females had been a victim of CV and felt some level of stress (*χ2* = 28.19, *p* < 0.001). PTSD symptoms increased along with an increase in CVE-related stress in both females and males, see Table 2. There was a weak correlation (ρ = 0.367; *p* < 0.001) between the two stress response measures in relation to CVE (i.e., by witnessing and victimization).

**Table 2:** Differences in self-assessed PTSDsymptoms (Mean (SD)) in relation to perceived stress during CVE, assessed with the Kruskal Wallis pairwise test

	PTSD symptoms in year 2					
	Females		Males		All	
	Mean (SD)	*n*	Mean (SD)	*n*	Mean (SD)	*n*
CVE Witnessed						
No CVE^a^	17.73 (11.94)	99	13.69 (10.91)	105	15.65 (11.58)	204
CVE and no stress^b^	19.81 (13.10)	36	17.23 (11.19)	71	18.09 (11.87)	107
CVE with little or some stress^c^	21.22 (13.04)	129	15.89 (12.02)	122	18.63 (12.81)	251
CVE with moderate or a lot of stress^d^	29.52 (17.58)	126	22.40 (14.49)	72	26.93 (16.84)	198
Total sample	22.88 (15.15)	390	16.79 (12.42)	370	19.91 (14.21)	760
Kruskal Wallis test	a < d***;b < d*; c < d**		a < d***; c < d*		a < c*; a < d***; b, c < d***	
CVE Victimization						
No CVE^a^	20.18 (13.42)	282	14.20 (11.58)	203	17.68 (13.01)	485
CVE and no stress^b^	22.52 (10.54)	23	20.00 (13.24)	42	20.89 (13.32)	65
CVE with little or some stress^c^	27.32 (16.05)	37	19.48 (13.01)	69	22.22 (14.56)	106
CVE with moderate or a lot of stress^d^	35.52 (18.74)	48	20.45 (12.16)	56	27.40 (17.21)	104
Total sample	22.88 (15.15)	390	16.79 (12.42)	370	19.91 (14.21)	760
Kruskal Wallis test	a < c*;a < d***		a < b*;a < c**; a < d***		a < c**; a < d***	

*CVE* Community violence exposure, *PTSD* posttraumatic stress disorder, *SD* Standard deviation

*p < 0.05. **p < 0.01. ***p < 0.001

### Associations between witnessing, its associated severity of stress and subsequent PTSD symptoms

The results from the GLM analysis for the association between witnessing violence at wave 1 and posttraumatic stress at wave 2 are presented in Table 3. Compared to those who had not witnessed violence, all those who witnessed CV with either no stress, or any amount of stress (from a little to a lot) had elevated PTSD symptom levels, with the posttraumatic stress levels being highest among those who had witnessed violence and had a moderate or a lot of stress. In addition, females had higher levels of PTSD symptoms than males. There was no significant effect for age, SES or ethnicity, and no interaction effect between sex and stress in relation to the effect of CVE on PTSD symptoms (*χ2* = 0.99, *p* = 0.804).

**Table 3:** Effect sizes on PTSDsymptoms of each study variable

	PTSD-symptoms in year 2
	beta (95% CI)
CVE Witnessed	
No CVE (Ref.)	
CVE and no stress	0.190 (0.026, 0.354)*
CVE with little or some stress	0.176 (0.046, 0.306)**
CVE with moderate or a lot of stress	0.485 (0.346, 0.624)***
Sex (female)	0.255 (0.154, 356)***
Age	0.069 (-0.008, 0.147)
SES	0.024 (-0.033, 0.081)
African American	− 0.137 (-0.293, 0.018)
Hispanic	− 0.081 (-0.262, 0.100)
CVE Victimization	
No CVE (Ref)	
CVE and no stress	0.274 (0.091, 0.456)**
CVE with little or some stress	0.295 (0.146, 0.444)***
CVE with moderate or a lot of stress	0.439 (0.290, 0.587)***
Sex (female)	0.353 (0.252, 0.455)***
Age	0.088 (0.011, 0.165)*
SES	0.015 (-0.042, 0.071)
African American	− 0.104 (-0.259, 0.051)
Hispanic	− 0.025 (-0.207, 0.156)

*CVE* Community violence exposure, *PTSD* posttraumatic stress disorder, *SES* Socioeconomic status, *Ref.* Reference category

*p < 0.05. **p < 0.01. ***p < 0.001

### Associations between victimization, its associated severity of stress and subsequent PTSD symptoms

As shown in Table 3, there was an effect for perceived stress severity in relation to CV victimization on PTSD symptoms, with those who reported victimization but no stress, a little or some stress, and a moderate amount or a lot of stress having elevated PTSD symptoms, when compared to those who reported no CVE. Females reported more PTSD symptoms than males, and age was positively associated with PTSD symptoms. Neither ethnicity nor SES were associated with PTSD symptoms at wave 2. There was no interaction effect between sex and stress during CVE on PTSD symptoms (*χ2* = 3.34, *p* = 0.343).

## Discussion

In this longitudinal study we found an association between the severity of the perceived stress associated with CVE and subsequent PTSD symptoms among U.S. adolescents. The students who reported CVE had higher PTSD symptom ratings than those who reported no CVE. The levels of future PTSD symptoms increased along with increasing levels of CVE-related stress severity one year prior, where experiencing a moderate amount to a lot of stress during CVE was especially strongly associated with elevated PTSD symptoms. The association between stress during CVE and PTSD symptoms was similar for adolescent females and males, although more females reported PTSD symptoms and the witnessing of CV with associated perceived stress, and more males reported being violently victimized with associated perceived stress.

As hypothesized, in this study population we found that those experiencing more stress during CVE also reported more PTSD symptoms at the follow-up. Although it should be noted that CVE was associated with higher PTSD symptoms even when no stress was reported in connection with the exposure, the levels of PTSD symptoms increased along with increased CVE-related stress at exposure, with the highest PTSD ratings (where the mean value indicated a moderate PTSD symptom level) reported by those who experienced a moderate amount or a lot of stress. Although the links between perceived stress during CVE and PTSD are relatively unexplored in adolescence, these findings are in line with those from an earlier study where a positive correlation was observed between CVE and PTSD, as well as between a general measure of perceived stress and PTSD [18] that was reported among children. Further, exposure to violence during adolescence has been shown to have an influence on future perceived stress throughout early adulthood [22]. These findings are further supported by the results from studies on adult populations that have investigated the relationship between perceived stress in relation to other types of trauma and PTSD, with reports of a positive association between general measures of perceived stress and PTSD amongst traumatized adults [19–21]. The present study further adds to the evidence that the levels of perceived stress severity during a traumatic event are important for the subsequent development of PTSD symptoms, where the subjective emotional response is of great importance for meeting the criteria for PTSD [12] and where subjective distress is independently associated with an increased risk for the development of PTSD symptoms [13].

Interestingly, the increase in PTSD symptoms when reporting a moderate amount or a lot of stress was similar among those who had witnessed or had been victimized by CV, hence not reflecting previous findings on the role of proximity to violence [10, 11] as a major factor in the negative outcomes associated with CVE. Rather, the subjective severity of stress seems to be of greater importance. Our findings support research that has linked perceived stress to negative health outcomes, including PTSD, where stress impacted on multiple biological systems which affect the regulation of the autonomic and neuroendocrine response to stressors [38]. From a theoretical perspective, cognitive theory highlights the importance of the interpretation of events, rather than the nature of the events themselves, in determining the consequences of those events for the individual [39]. Indeed, according to the cognitive-processing theory of PTSD, a traumatic event will be harder to process or assimilate with prior knowledge if the individual becomes highly stressed and overwhelmed, which is why psychological defense mechanisms are brought into play in order to manage the distress, including denial of the trauma, numbing, flashbacks and nightmares [39]. Ehlers and Clark’s cognitive model of PTSD also stresses the importance of the individual’s appraisal of the trauma, given that a negative appraisal of the meaning of the trauma can sometimes create a sense of a serious current threat, and an individual can also experience impaired memory encoding during the trauma where the traumatic memory becomes fragmented and poorly integrated into autobiographic memories [40].

In accordance with previous research, we found that PTSD symptoms were more common amongst females [41–44]. It has been suggested that this disparity can be potentially explained by the sex differences in relation to coping skills, as well as neurobiology [45]. Earlier research has suggested that females have heightened stress-sensitivity with a more sensitive HPA-axis, and as a result of this, an increased risk of developing stress-related conditions such as PTSD [45]. When comparing perceived stress in relation to CVE between the sexes, more females than males reported witnessing CV and feeling a moderate amount or a lot of stress, whereas more males than females reported being victimized by CV and feeling any level of stress. Previous research has also reported that males are more likely to be exposed to violence [18, 46, 47], and more females than males have reported witnessing CV [33, 34]. In contrast to what was hypothesized, this study found no interaction between sex and CVE-related stress on PTSD symptoms, suggesting that the relationship between perceived stress during CVE and later PTSD symptoms is independent of sex. Although the results were not as expected, this underlines the complexity of the issues involved in the development of PTSD.

Our study has several strengths, including the use of a relatively large sample with an even sex distribution, and being able to apply a longitudinal study design. Nonetheless, there are several limitations that should be mentioned. This study was carried out with data from a population of urban youth with a high proportion of ethnic minority adolescents and low SES, limiting the generalizability of our study findings. The use of self-reported data may increase the risk of recall and reporting bias, with males being less likely to report their mental health problems [48]. Therefore, being able to use other data sources such as information that was collected for example, during a clinical interview that used the Clinician-Administered PTSD Scale (CAPS-5) [49] would have been desirable. Further, the CPTS-RI is based on earlier DSM versions, and not on DSM-5 which has an increased focus on behavioral/cognitive symptoms and includes alterations in the symptom clusters [4]. Although the CPTS-RI encompasses the core criteria for PTSD including re-experiencing, avoidance/numbing and arousal, a measure that corresponded to the latest version of PTSD symptoms listed in DSM-5 and that also included more questions targeting alterations in mood and cognition would have provided additional information. The study did not consider the number of times each student had been exposed to CV, and did not include other relevant traumas such as sexual abuse, which could have affected the results. For instance, many of those with no CVE reported mild PTSD symptom levels, which could be related to other traumas not measured by this study. Moreover, we did not include information regarding whether the students had experienced PTSD symptoms at baseline which would have provided evidence on how the symptoms might fluctuate across adolescent development. In addition, when assessing CVE-related stress, some students may have reported no stress in relation to witnessing violence but stress in relation to experiencing violent victimization, and vice versa, and as such, been coded as no CVE in one of the models and as exposed to CVE in the other. At the same time the correlation between the two stress response measures in relation to CVE (i.e., by witnessing and victimization) was weak. We also lacked data on other potentially traumatic events, or any contact with authorities, that may have occurred between the two data collection waves. In addition, we had no information on whether the students had been diagnosed with or were receiving treatment related to PTSD or other psychiatric conditions during the study period, factors that might have affected the levels of PTSD symptoms. Importantly, we also had no data on protective factors such as social support, which could have been important for the observed associations. Although the study was able to show a longitudinal association between the levels of perceived stress severity during CVE and future PTSD symptoms, causality could not be established. Finally, the data were collected two decades ago and although we have no reason to believe that the observed associations would change had the data been collected more recently, as the prevalence of both forms of CVE and PTSD symptoms could have changed with time, there is a need for future research on these associations with more recent data.

In conclusion, this is the first longitudinal study to suggest that the levels of CVE-related stress may affect future PTSD symptoms in adolescents, regardless of sex. Identifying those who are at an increased risk of developing PTSD from among all those individuals that are exposed to CV is a critical task for early screening, and these findings underline the potential role of perceived stress severity in relation to CVE as a possible factor that can be used for identifying those at risk. Hence, interventions should focus on adolescents who report higher levels of perceived stress severity during CVE, as they may be at increased risk of developing PTSD symptoms in the future. Such interventions may potentially include therapeutic approaches such as trauma-focused cognitive behavioral therapy (TF-CBT) [50]. Further, given the potential importance of the subjective stress experienced in relation to the exposure, providing immediate psychological support to adolescents exposed to CV, including the use of stress reduction techniques such as mindfulness, relaxation exercises, or emotional support, might be extremely valuable when it comes to preventing CVE-related mental health problems in adolescents. In terms of future research, more longitudinal studies on large general population samples are needed in order to better understand the relationship between perceived stress, community violence and PTSD in youths, that also include baseline symptoms and data sources other than self-reports.

## Data Availability

No datasets were generated or analysed during the current study.

## Abbreviations

CAPS-5: Clinician-administered PTSD scale
CPTS-RI: Child post-traumatic stress reaction index
CV: community violence
CVE: Community violence exposure
DSM: Diagnostic and statistical manual of mental disorders
PTSD: posttraumatic stress disorder
SES: socioeconomic status
